# Lateral radial head offset can be associated with chronic epicondylopathia humeri radialis: A new radiological parameter

**DOI:** 10.1002/ksa.70410

**Published:** 2026-04-16

**Authors:** Romed P. Vieider, Sebastian Siebenlist, Alexander Wolfgang Marka, Lukas N. Muench, Lucca Lacheta, Franziska Rudolph, Stephanie Geyer, Pavel Kadantsev

**Affiliations:** ^1^ Department of Sports Orthopaedics TUM University Hospital, Technical University of Munich Munich Germany; ^2^ Institute for Diagnostic and Interventional Radiology, School of Medicine and Health, TUM Klinikum Technical University of Munich (TUM) Munich Germany; ^3^ Department of Traumatology University Hospital Zurich, University of Zurich Zurich Switzerland; ^4^ St. Vinzenz Clinic Pfronten Pfronten Germany

**Keywords:** arthroscopic debridement, arthroscopy, chronic epicondylitis, epicondylopathia humeri radialis, posterolateral instability, radial head

## Abstract

**Purpose:**

This study aimed to compare anatomical parameters on magnetic resonance imaging (MRI) scans between patients with symptomatic isolated chronic epicondylopathia humeri radialis (cER) and healthy controls. It was hypothesized that the radial head would show an increased lateral offset in patients with cER.

**Methods:**

This retrospective radiological case–control study included patients with cER (symptomatic > 6 months) from November 2020 to July 2023. The MRIs of all included patients were compared to those of healthy controls. Radiological measurements were performed in millimetres (mm): lateral humeral epicondyle prominence, radial head diameter, lateral radial head offset, lateral radial head offset ratio (lateral radial head offset/radial head diameter; %) and posterior radial head translation.

**Design:**

Retrospective radiological case–control study.

**Results:**

Seventy‐seven elbows (37 cER/40 healthy controls) from 57 patients (37 male; age: 39.3 ± 11.2 years; body mass index: 23.0 ± 7.6) were included. There was no significant difference between groups in lateral humeral epicondyle prominence (11.7 ± 8.8 mm vs. 11.0 ± 1.6 mm, *p* = 0.051) or radial head diameter (22.2 ± 4.1 mm vs. 22.2 ± 3.7 mm, *p* = 0.779). Lateral radial head offset was significantly greater in the cER group compared with healthy controls (mean difference: 1.84 mm, 95% confidence interval [CI] = 1.07–2.60; *p* < 0.001), while the lateral radial head offset ratio did not differ significantly (21.1% vs. 9.1%, *p* = 0.691). Posterior radial head translation was also significantly increased in the cER group (mean difference: 4.35 mm, 95% CI = 3.72–4.98; *p* < 0.001). There was no significant difference in ulnohumeral incongruence between groups (2.2 ± 0.6 mm vs. 2.4 ± 0.6 mm, *p* = 0.238).

**Conclusion:**

Patients with cER showed significantly higher lateral radial head offset and posterior radial head translation on MRI compared to healthy controls. This may suggest that the dynamic stabilizers are weakened in cER patients, resulting in a relative shift of the radial head towards the lateral and posterior position.

**Level of Evidence:**

Level III.

**Abbreviations**
BMI: body mass index
cER: chronic epicondylopathia humeri radialis
mm: millimetre
MRI: magnetic resonance imaging
n.s.: non‐significant
PLRI: posterolateral rotatory instability
SD: standard deviation

## INTRODUCTION

Chronic epicondylopathy humeri radialis (cER), formerly often referred to as ‘tennis elbow’, is the most common cause of elbow pain. In the majority of cases, it is a self‐limiting condition, with most patients becoming free of symptoms within 1 year after the initial onset [5, 15, 20, 22]. In 4%–11% of patients, symptoms persist despite adequate conservative treatment, leading to significant limitations in daily life and requiring surgical treatment [15, 23]. Defining predictive factors of cER that require surgical treatment remains under investigation.

There is evidence that persistent cER leads to chronic inflammatory and degenerative changes with progressive weakening of the lateral capsuloligamentous complex [11, 13, 14]. Consequently, increased posterior translation of the radial head in patients with cER was associated with pathological changes in the lateral stabilizers beyond subclinical radiocapitellar incongruence. This results in posterolateral rotatory instability (PLRI) of the humeroradial joint [2, 13].

It is therefore important to identify patients who develop cER early, and various risk factors have been suggested to contribute to this condition, such as female sex, smoking history, heavy labour, repetitive use of the common extensor mechanism or mental conditions such as depression [10, 21, 25]. The anatomical risk factors that predispose patients to developing cER have rarely been investigated. This study proposes a measurement technique that quantifies lateral radial head offset in relation to the insertion of the common extensor tendon of the humerus. It was hypothesized that patients with cER experience increased lateral radial head offset due to inadequate dynamic muscular stabilization, resulting in increased stress on the lateral joint capsule from the lateralization of the radial head.

Therefore, the aim of this study is to introduce a measurement method to assess the lateralization of the radial head with respect to the humerus, which is introduced as the lateral radial head offset. It was hypothesized that cER would lead to a higher lateral radial head offset compared to healthy controls.

## MATERIALS AND METHODS

This was a retrospective radiological study approved by the Ethics Committee of the Technical University of Munich (No. 2023‐452‐S‐SB). All consecutive patients with cER (symptomatic > 6 months) who failed conservative treatment and underwent arthroscopic debridement of the extensor carpi radialis brevis at a single institution from November 2020 to July 2023 were included in this study. Patients with previous bony or musculoligamentous injuries, previous surgeries or invasive treatment such as any kind of injections on their elbow joint were excluded. Furthermore, patients with unavailable preoperative magnetic resonance imaging (MRI), low MRI scan quality or MRI not taken in the neutral position (full extension) were excluded. If there were clinical medial or lateral instability (positive drawer test, prone push‐up test and lateral pivot shift test), radiological instability (ligamentous lesions, chondral lesions, intraarticular loose bodies), patients were excluded. All patients in the cER group underwent standardized arthroscopic evaluation of all compartments. The presence of a positive drive‐through sign, indicating PLRI, was an exclusion criterion [18].

All patients in the cER group underwent standardized arthroscopic evaluation of all elbow compartments to assess for a positive drive‐through sign indicating PLRI; patients with confirmed PLRI were excluded [18].

### Measurements

The MRI scans of all included patients were compared to an existing dataset of healthy controls, which was used in a previous radiological study [8]. All MRIs were performed in full elbow extension (0°) and supination (90°) on the same scanner using a standardized protocol (3D proton density–weighted sequence with spectral attenuated inversion recovery [SPAIR], sagittal acquisition), with a slice thickness of 3 mm for all patients. This sequence allowed for multiplanar reconstructions, enabling adjustment of imaging planes when the original acquisition was not perfectly aligned. All images were analyzed the institutions' Picture Archiving and Communication System (PACS, Sectra Medical Systems).

**Figure 1:**
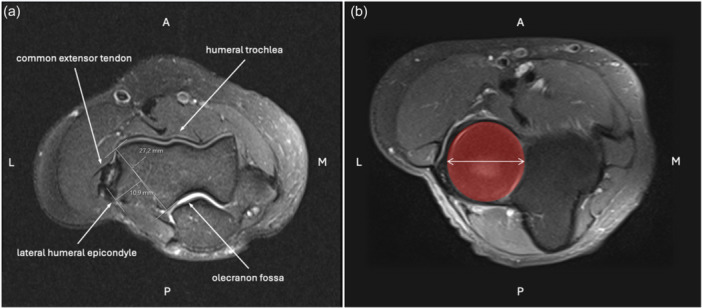
Measurement of the lateral epicondyle prominence (a) was measured as the perpendicular distance from the most prominent point of the lateral epicondyle to a line connecting the proximal anterior (lateral trochlea humeri) and posterior (lateral fossa olecrani) borders of the lateral humeral cartilage in the axial sequence in millimetres. The radial head diameter (b, white arrow) was measured in millimetres in the first axial sequence, where the radial head was fully depicted (red circle).

To determine the lateral epicondyle prominence, a line was drawn connecting the most proximal points of the anterior (lateral trochlea humeri) and posterior (lateral fossa olecrani) borders of the lateral humeral cartilage in the axial sequence. A perpendicular line was then drawn to the most prominent point of the lateral epicondyle, and the distance between these lines was measured in mm (Figure 1a).

**Figure 2:**
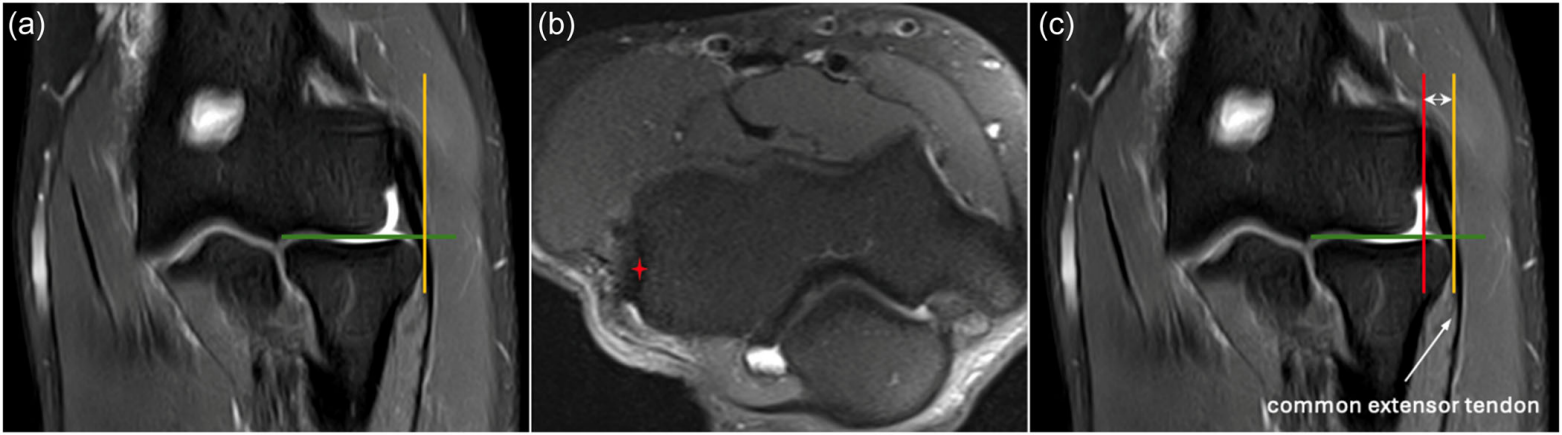
Measurement of the lateral radial head offset. (a) The centre of the radial head was identified in the coronal plane, and a tangent line was drawn along its joint surface as a reference (green line). A perpendicular line was then placed at the most lateral point of the radial head (yellow line). (b) Next, the centre of the common extensor tendon origin was located on the lateral condyle (red asterisk) in the axial plane, and the corresponding coronal plane was determined. (c) A second perpendicular line was drawn through this centre on the coronal plane (red line), and the distance between the two parallel lines (red and yellow) was measured in millimetres (white arrow).

The radial head diameter was measured in the first axial sequence, which fully depicted the radial head (Figure 1b). To determine the lateral offset of the radial head relative to the common extensor origin, the following steps were performed: First, the coronal plane of the centre of the radial head was defined, and a tangent line was drawn along the joint surface of the radial head. A perpendicular line to the humeroradial joint line was then projected, tangent to the most lateral part of the radial head (Figure 2a). Second, the centre of the origin of the common extensor tendon was identified on the lateral condyle of the humerus in the axial plane (Figure 2b). Once this centre was defined, the corresponding coronal plane was identified using the localizer tool. In this plane, a second perpendicular line was drawn from proximal to distal, intersecting the centre of origin of the common extensor tendon, which was previously defined on the axial plane. The distance between the two lines perpendicular to the humeroradial joint line (white double arrow) was measured in mm (Figure 2a). The lateral radial head offset was defined as an absolute distance (mm) to reflect the spatial relationship between the lateral border of the radial head and the common extensor tendon footprint. The lateral radial head offset ratio (lateral radial head offset/radial head diameter; %) was calculated to express the lateralization of the radial head relative to its diameter.

**Figure 3:**
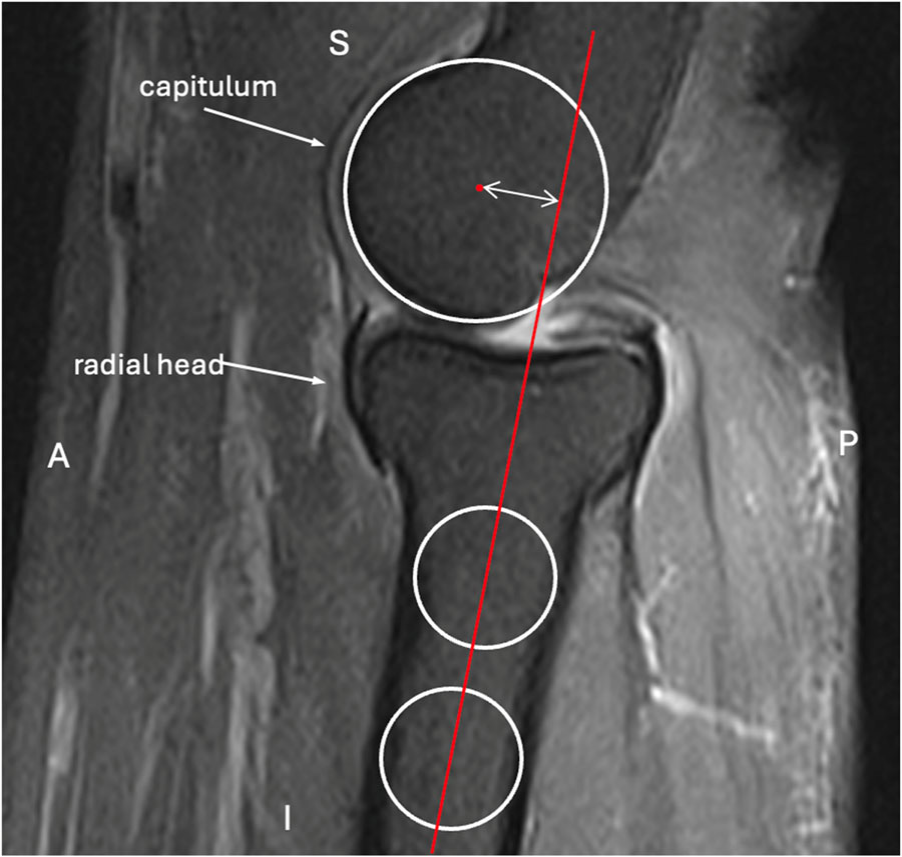
The posterior radial head translation was defined as the distance between the centre of the capitellum (red dot) and the radial shaft axis (red line) in millimetres, according to Hackl et al. [9].

Furthermore, the width of the capitellum was measured in the same coronal plane in which the centre of the radial head was determined (Figure 3a). The posterior radial head translation was determined according to a previously established method by Hackl et al. [9] (Figure 3).

All radiological measurements were performed by three independent, blinded raters: two experienced sports‐orthopaedic fellows (R.P.V. and P.K.) and one radiologist with expertise in musculoskeletal radiology (A.M.).

### Statistics

An a priori power analysis was performed using G*Power v.3.1.9.7 [7]. Based on a pilot sample of ten elbows, the observed mean difference in lateral radial head offset between cER patients and healthy controls was 1.5 ± 1.4 mm, corresponding to an effect size of *d* = 1.13. Assuming an alpha level of 0.05 and a power of 80% for an independent‐samples *t* test, a total sample size of 38 elbows (19 per group) would be required. Accounting for a potential attrition rate of 10%, a target sample size of 21 elbows per group was considered sufficient.

Due to the expected difference in age of both groups (predefined control group), a sensitivity analysis was performed using linear regression models adjusting for age to assess whether group differences in the main outcome parameter (lateral radial head offset) were independent of age.

Descriptive data on measurements were displayed in graphs and tables. Normal distribution was assessed using the Kolmogorov–Smirnov test. In case of normally distributed data, the Student's *t* test, in case of non‐normally distributed data, the Mann–Whitney *U* test was used for group comparisons. The level of significance was set at *p* < 0.05. Interrater agreement between three raters was measured using the intraclass correlation coefficient (ICC); 0–0.2 represents slight agreement; 0.21–0.4 indicates fair agreement; 0.41–0.6 signifies moderate agreement; 0.61–0.8 shows substantial agreement; and 0.81–1.0 indicates almost perfect agreement [1].

## RESULTS

### Demographic data

A total of 37 patients with cER and 40 healthy controls were included in the study. The mean age of the cER group was significantly higher (48.2 ± 7.6 years) compared to the healthy controls (31.3 ± 7.0 years, *p* < 0.001). There were no significant differences in sex distribution and body mass index between the groups (Table 1).

**Table 1:** Demographics and comparison of measurements of patients with chronic epicondylopathia humeri radialis (cER) versus healthy controls, number of patients (*n*), years (a), standard deviation (SD), millimetre (mm), body mass index (BMI) and non‐significant (n.s.).

Parameter	cER	Healthy controls	*p*
*n*	*n* = 37	*n* = 40	n.s.
Age (a)	48.2 ± 7.6	31.3 ± 7.0	***p*** < **0.001**
Sex			
Male, *n* (%)	17 (45.9%)	20 (50%)	n.s.
Female, *n* (%)	20 (54.1%)	20 (50%)	n.s.
BMI	25.5 ± 4.1	24.3 ± 8.4	n.s.
Measurements			
Lateral epicondyle prominence	11.8 ± 6.2	11.8 ± 1.6	n.s.
Radial head diameter (mm ± SD)	22.4 ± 4.1	22.2 ± 3.7	n.s.
Lateral radial head offset (mm ± SD)	3.3 ± 1.2	1.5 ± 2.1	***p*** < **0.001**
Lateral radial head offset ratio^a^ (%, range)	21.1% (3.7–27.9)	9.1% (4.2–28.4)	n.s.
Posterior radial head translation (mm ± SD)	5.1 ± 1.5	0.7 ± 1.2	***p*** < **0.001**
Humeroulnar incongruence (mm ± SD) (mean of four measurements)	2.1 ± 0.7	2.2 ± 0.7	n.s.

*Note*: Bold values indicate statistically significant.

a Lateral radial head offset (mm)/radial head diameter (mm).

### Measurements

**Figure 4:**
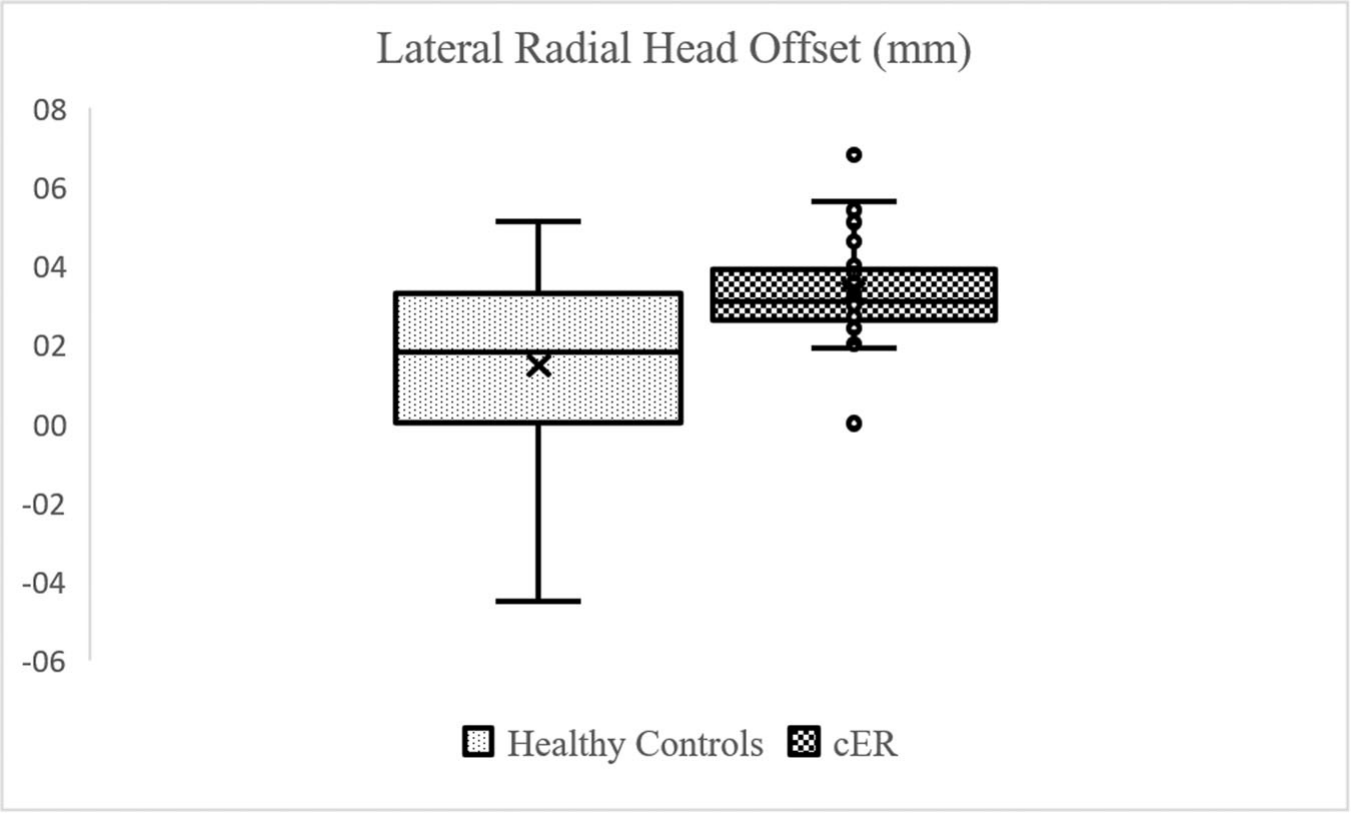
Boxplot diagram showing the difference in lateral offset of the radial head of healthy controls versus patients with chronic epicondylopathy humeri radialis (cER) in millimetres (mm). Statistical significance (*p < 0.05).

**Figure 5:**
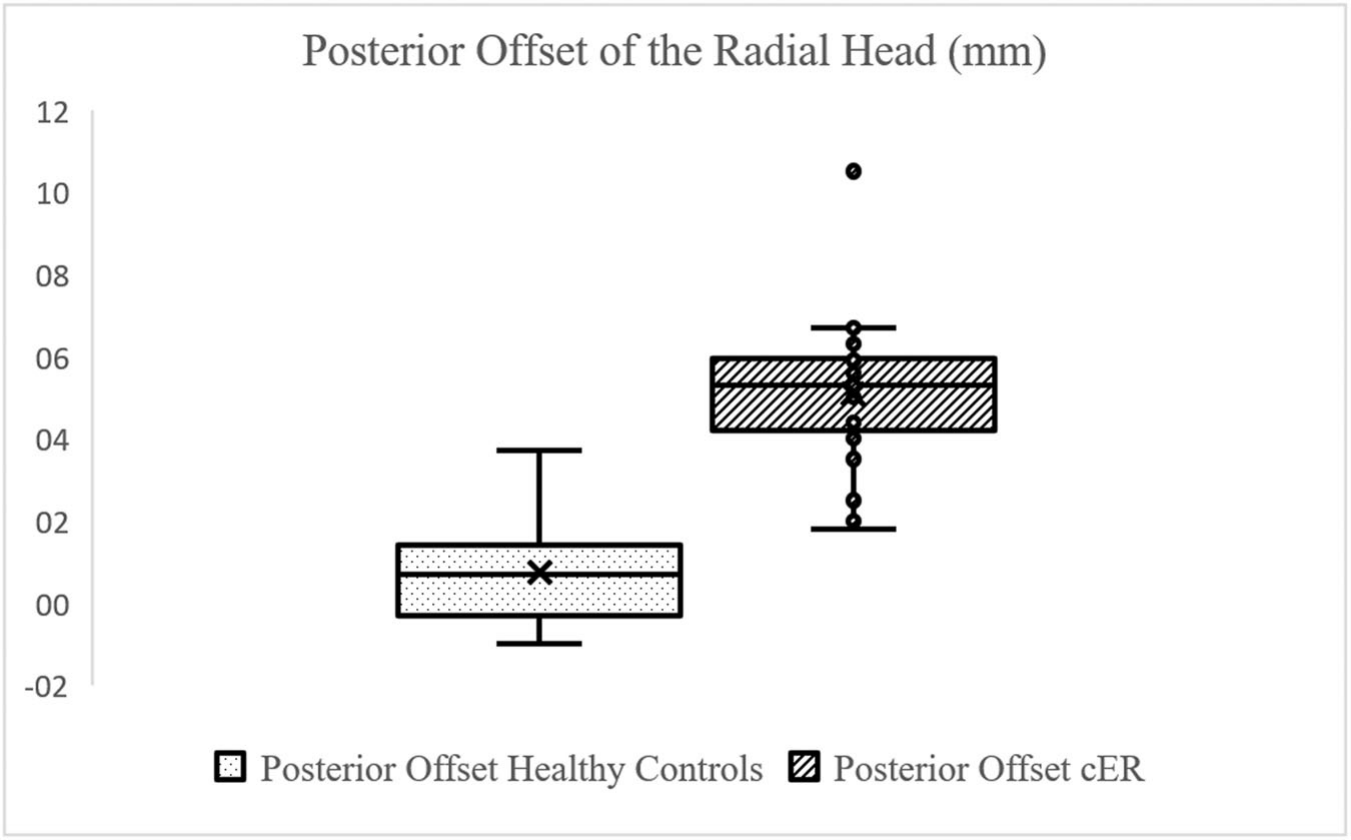
Boxplot diagram showing the difference in the posterior radial head translation (mm) of healthy controls versus patients with chronic epicondylopathy humeri radialis (cER) in millimetres (mm). Statistical significance (*p < 0.05).

The lateral epicondyle prominence was similar in both groups, with a mean of 11.8 ± 6.2 mm in the cER group and 11.8 ± 1.6 mm in the control group (*p* > 0.05). Radial head diameter also showed no statistically significant difference between groups (22.4 ± 4.1 mm in the cER group vs. 22.2 ± 3.7 mm in controls, *p* > 0.05). In contrast, the lateral radial head offset was significantly greater in the cER group (3.3 ± 1.2 mm) compared with healthy controls (1.5 ± 2.1 mm, mean difference: 1.84 mm, 95% CI: 1.07–2.60; *p* < 0.001) as displayed in Figure 4. After adjustment for age, lateral radial head offset remained significantly greater in the cER group compared with healthy controls (*B* = 2.04 mm, 95% CI: 0.83–3.24; *p* = 0.001). Age was not a significant predictor (*p* = 0.662). The lateral radial head offset ratio was not significantly different between groups (cER: 21.1% [range: 3.7–27.9] vs. control: 9.1% [range: 4.2–28.4]). Posterior radial head translation was significantly higher in the cER group (5.1 ± 1.5 mm) than in controls (0.7 ± 1.2 mm, *p* < 0.001; mean difference: 4.35 mm, 95% CI: 3.72–4.98; *p* < 0.001) as displayed in Figure 5. Finally, humeroulnar incongruence, calculated as the mean of four measurements, did not differ significantly between the cER group (2.1 ± 0.7 mm) and controls (2.2 ± 0.7 mm, *p* > 0.05). ICCs indicated moderate to substantial reliability for all measurements as displayed in Table 2.

**Table 2:** Intraclass correlation coefficients (ICC) of the measurements and the corresponding 95% confidence intervals (CIs).

Interrater reliability of measurements			
Variable	ICC	95% CI	
Lateral epicondyle prominence	0.906	0.85	0.94
Radial head diameter	0.71	0.53	0.83
Lateral radial head offset	0.71	0.53	0.83
Posterior radial head translation	0.76	0.61	0.86
Humeroulnar incongruence (mean of four measurements)	0.69	0.54	0.86

## DISCUSSION

The main finding of this study was that patients with cER exhibited a significantly increased lateral radial head offset and increased posterior radial head translation compared to healthy controls. This supports the hypothesis that radiocapitellar joint incongruency in patients without posterolateral instability or hyperlaxity may be indicative of cER.

The current study demonstrated an increased lateral offset and increased posterior translation of the radial head in patients with cER. These findings may reflect weakening of the anterior and lateral joint capsule, potentially driven by a persistent inflammatory stimulus and associated lateralization of the radial head. Given the ongoing debate regarding the aetiology of cER and the wide range of proposed risk factors, such as repetitive loading of the extensor mechanism, heavy manual labour, smoking, and psychological factors, including depression, anatomical risk factors that may predispose to cER have been comparatively underinvestigated [4, 10, 21, 25]. The anterior extensor carpi radialis brevis origin is a fragile structure due to its purely tendinous structure and thin capsular attachment compared to its posterodistal region, potentially predisposing it to lateral cER [16]. Another study indicated that a compensatory mechanism from cER may result in an increased ulnohumeral angle [24]. This change in the ulnohumeral angle may contribute to a changed position of the radial head due to the altered joint kinematics. The current study describes the relationship between the radial head and the insertion of the common extensor origin and may indicate a change in the position of the radial head due to compensatory joint kinematics.

The influence of muscles as dynamic stabilizers on joint congruency in the development of cER has been investigated by a retrospective analysis of MRIs from 160 cER patients [13]. That study demonstrated an association between the severity of pathological changes in the extensor tendons and radiocapitellar incongruency [13]. These findings are consistent with the results of the present study and support an association between cER and altered elbow congruency. Using the lateral radial head offset measurement, the present study provides additional imaging evidence of altered bony relationships within the joint. These findings are compatible with the hypothesis that capsuloligamentous elongation and insufficiency of dynamic stabilizers may contribute to radiocapitellar incongruence, underscoring the multifactorial nature of cER, particularly in symptomatic and therapy‐resistant cases.

It is important to note that there is currently no consensus on the definition of physiological elbow joint congruence in MRI. Previous research has shown that joint congruence depends on both its position and inherent laxity, as seen in the current study [3, 12, 17, 19]. A significantly higher radiocapitellar joint incongruency in patients with PLRI was reported [9]. In a cadaveric study, a high variability in the mean lateral joint opening (4.0 ± 1.2 mm) was observed in healthy elbows during arthroscopic rod stability testing [18]. The lateral joint opening is particularly relevant, considering that cadaveric models account only for static stabilizers, highlighting the variability of the joint capsule laxity. In the current study, all patients who underwent arthroscopic debridement of the common extensor origin were tested to determine whether hyperlaxity was present, using a previously published method with a rod [18]. Nevertheless, there was a significantly higher lateral offset and posterior translation of the radial head with respect to the capitellum.

Given that none of the patients in the current study had clinical, intraoperative, or additional MRI evidence of posterolateral instability or hyperlaxity, it was hypothesized that the increased lateral offset and posterior translation resulted from a secondary elongation of the capsuloligamentous complex. The elongation of the capsule may occur due to the constant inflammatory process in the region of the lateral and posterior radiocapitellar joint. Furthermore, muscle weakness in cER could lead to a loss of dynamic stabilization of the radial head, which could result in increased chronic stress on the posterolateral capsule. Finally, chronic overuse may put the lateral collateral ligament complex under constant tension and strain. This may further lead to degeneration of the lateral collateral ligament complex and development of PLRI. A recent case report showed that the so‐called ‘cam‐effect’ due to a radial head deformity may contribute to decentration of the humeroradial column and progressive elongation of the lateral ulnar collateral ligament complex, leading to recurrence of PLRI [6].

This study provides further insight into the pathogenesis of cER. Specifically, the focus is on the lateral radial offset, and the posterior translation may serve as an early indicator of lateral stabilizers pathology. The clinical value remains limited given the high variability in joint laxity and the complex anatomy of the elbow; therefore, further studies are required to define normal joint congruence and to establish reliable cut‐off values for incongruence measurements.

It is important to note that this study has several limitations: Its retrospective design resulted in a lack of standardization in the MRI protocol for the cER group. To mitigate this, all MRIs were assessed for quality, magnetic field strength (≥3 T) and joint positioning. Patients with unsatisfactory MRI quality were excluded. Another limitation may be the positioning of the elbow joint during imaging. All MRIs were performed in supination and extension, which was previously identified as causing maximal joint incongruity. This factor was controlled for by selecting MRIs of healthy patients from our database with the same joint position. However, the highly standardized MRI protocol in the control group versus the cER group may alter results. Finally, the significant age difference between the two groups must be considered. In addition to the absence of significant age‐related joint degeneration in the cER group, the sensitivity analysis did not indicate an age‐related confounder. However, age can still be considered a potential confounding factor. Finally, as this study was designed to identify diagnostic imaging parameters rather than to assess treatment outcomes, correlations with clinical scores or postoperative results were beyond its scope and should be addressed in future prospective studies.

## CONCLUSION

The lateral radial head offset and posterior radial head translation were significantly higher in patients with cER compared to healthy controls on MRI. This finding indicates a relative lateral and posterior decentring of the radial head relative to the humerus, which may be associated with chronic degenerative weakening of the surrounding stabilizers.

## AUTHOR CONTRIBUTIONS

**Romed Vieider**: Conceptualization; methodology; writing—original draft; writing—review and editing. **Stephanie Geyer**: Conceptualization; formal analysis; investigation; writing—original draft. **Sebastian Siebenlist**: Conceptualization; writing—review and editing. **Pavel Kadantsev**: Conceptualization; methodology; formal analysis; investigation; writing—original draft; writing—review and editing. **Lucca Lacheta**: Methodology; writing—review and editing. **Alexander Marka**: Methodology. **Lukas N. Muench**: Formal analysis; investigation; writing—original draft. **Franziska Rudolph**: Formal analysis; investigation; writing—original draft.

## CONFLICT OF INTEREST STATEMENT

Sebastian Siebenlist has received consultant fee payments from Arthrex GmbH, KLS Martin Group and medi GmbH & Co.KG unrelated to this study. The remaining authors declare no conflicts of interest.

## ETHICS STATEMENT

The authors have nothing to report.

## Data Availability

The data that support the findings of this study are available on request from the corresponding author. The data are not publicly available due to privacy or ethical restrictions.
